# Scrotal Rejuvenation

**DOI:** 10.7759/cureus.2316

**Published:** 2018-03-13

**Authors:** Philip R Cohen

**Affiliations:** 1 Department of Dermatology, University of California, San Diego

**Keywords:** alopecia, angiokeratoma, genital, hypertrichosis, laxity, rejuvenation, scrotal, scrotum, vaginal, wrinkling

## Abstract

Genital rejuvenation is applicable not only to women (vaginal rejuvenation) but also to men (scrotal rejuvenation). There is an increased awareness, reflected by the number of published medical papers, of vaginal rejuvenation; however, rejuvenation of the scrotum has not received similar attention in the medical literature. Scrotal rejuvenation includes treatment of hair-associated scrotal changes (alopecia and hypertrichosis), morphology-associated scrotal changes (wrinkling and laxity), and vascular-associated scrotal changes (angiokeratomas). Rejuvenation of the scrotum potentially may utilize medical therapy, such as topical minoxidil and oral finasteride, for scrotal alopecia and conservative modalities, such as depilatories and electrolysis, for scrotal hypertrichosis. Lasers and energy-based devices may be efficacious for scrotal hypertrichosis and scrotal angiokeratomas. Surgical intervention is the mainstay of therapy for scrotal laxity; however, absorbable suspension sutures are postulated as a potential intervention to provide an adequate scrotal lift. Hair transplantation for scrotal alopecia and injection of botulinum toxin into the dartos muscle for scrotal wrinkling are hypothesized as possible treatments for these conditions. The interest in scrotal rejuvenation is likely to increase as men and their physicians become aware of both the conditions of the scrotum that may warrant rejuvenation and the potential treatments of the scrotum for these individuals.

## Introduction and background

The scrotum covers the testicles, epididymis, vas deferens, and the external spermatic fascia along with the lower spermatic cords, in addition to accompanying arteries, veins, lymphatics, and nerves. Changes occur in the integrity of scrotum as men age. This paper discusses scrotal skin changes and the potential interventions for rejuvenation of the scrotum.

## Review

Genital rejuvenation

Skin alterations in the genital region occur with age. These may be secondary to intrinsic or extrinsic etiologies. The morphologic and functional changes that occur may create medical conditions or aesthetic concerns or both for the affected individuals.

Vaginal rejuvenation

The genital region in women includes the mons pubis, the vulva (containing the clitoris, labia majora, and labia minora), and the vagina. Rejuvenation of the female genital region has been referred to as vulvovaginal rejuvenation or, more commonly, as vaginal rejuvenation.

Interest in rejuvenation of the female genital region—measured by the number of indexed papers on the subject—is recent and rapidly growing. When the PubMed search engine (which accesses citations from the MEDLINE database of biomedical literature) was used on February 15, 2018 for the term “vaginal rejuvenation” and “vulvovaginal rejuvenation,” 44 relevant articles were found. The first paper was published by the American College of Obstetricians and Gynecologists Committee on Gynecologic Practice in 2007 [1]. Subsequently, only a single paper is listed from 2010 and 2011; yet, beginning in 2012 through 2014, four papers were published each year. However, since 2015, 29 papers have been indexed: 11 in 2015, eight in 2016, and 10 in 2017.

Scrotal rejuvenation

Cosmetic concerns and procedures are increasing in men [2-3]. Indeed, aging can result in scrotal changes, such as laxity presenting with a low-hanging scrotum. Therefore, aesthetic and functional restorations of the scrotum are important aspects of scrotal rejuvenation [4]. However, to date, the term “scrotal rejuvenation” does not elicit any relevant citations using the PubMed search engine. 

Scrotal anatomy

Embryologically, the scrotum in men is analogous to the labia majora in women. Both originate from the labioscrotal swelling. Sex-appropriate development occurs after approximately the ninth week of gestation [5].

The scrotum is a sac consisting of two lobes that extend downward from the perineal region; it is located posterior to the penis. The median raphe is a linear thickening that defines where the lobes meet. The raphe extends anteriorly from the anus to the posterior surface of the scrotum, around the scrotum to its anterior surface, and then onto the ventral surface of the penis [5].

The scrotum consists of skin with its associated adnexal structures (such as hair) and the dartos tunic. The latter is composed of the smooth muscle and fascia. Ridges and furrows usually define the external wrinkled cutaneous surface of the scrotum [5].

Hair-associated scrotal changes

Scrotal Alopecia

The normal density of hair on the scrotum, to the best of my knowledge, has not been quantified; it is likely to vary based on age. In addition, a race-associated variability of hair density on the mons pubis and labia majora has been observed in women; in particular, investigators have noted a racial predisposition for pubic atrichosis or hypotrichosis in Korean women of Mongolian origin [6]. Therefore, it is reasonable to postulate that a racial predisposition for scrotal alopecia also exists.

**Figure 1:**
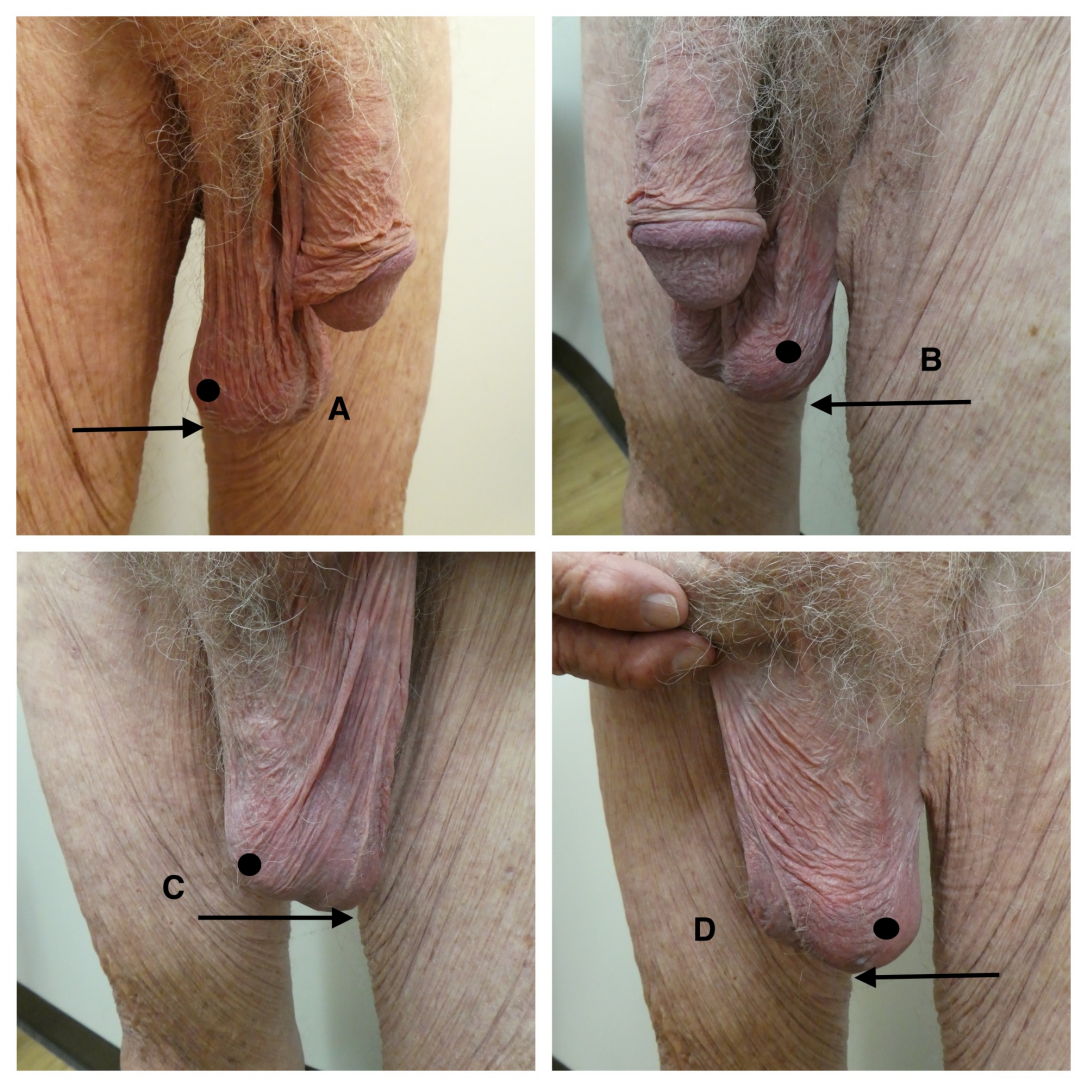
Scrotal alopecia and scrotal laxity in a 93-year-old man Distant (A and B) views of the penis and scrotum and closer (C and D) views of the scrotum demonstrate scrotal alopecia and severe scrotal laxity. The right side (A and C) and left side (B and D) of the scrotum (circle) show nearly complete absence of hair and bilateral low-hanging scrotum that extend to the mid-thigh (arrows).

**Figure 2:**
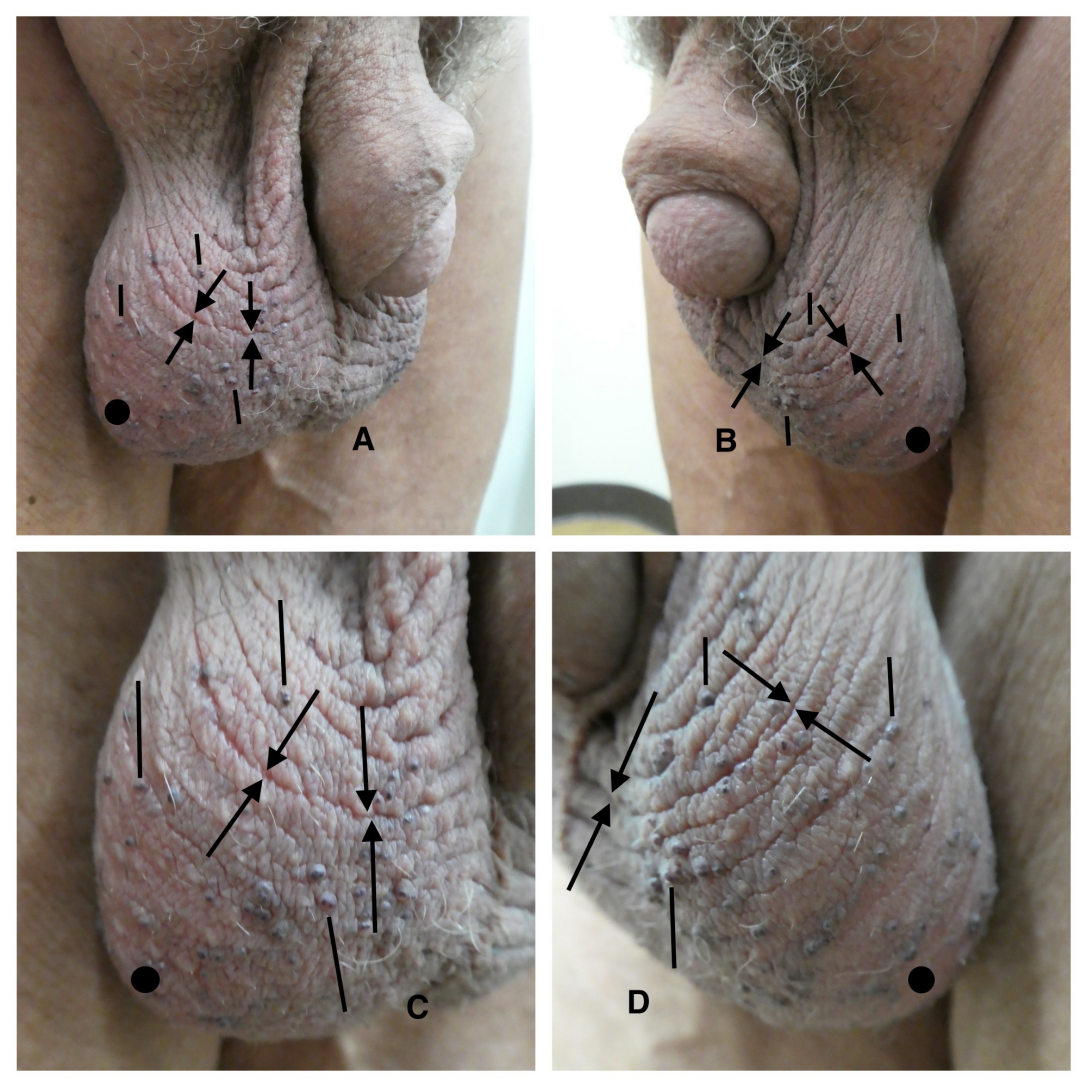
Scrotal alopecia, scrotal wrinkling, and scrotal angiokeratoma in a 63-year-old man Distant (A and B) views of the penis and scrotum and closer (C and D) views of the scrotum demonstrate scrotal alopecia, scrotal wrinkling, and scrotal angiokeratoma. The right side (A and C) and left side (B and D) of the scrotum (circle) show nearly complete absence of hair. Scrotal wrinkling (scrotum rugosum) is demonstrated by the prominent ridges and furrows (between the arrows). Numerous scrotal angiokeratomas, presenting as purple vascular papules (at either the inferior or superior tips of the solid bars), are shown on both sides of the scrotum.

Acquired alopecia of the scrotum may be idiopathic or possibly age-related (Figures 1, 2). Alternatively, it may be associated with repetitive friction from rubbing by the individual’s clothing. Hair loss can be caused by the grooming practices of the man; these could include epilation (by plucking or waxing) or removal by laser and other energy devices. Medication-related hair loss during treatment with systemic chemotherapy or targeted therapy, such as vismodegib for basal cell carcinoma, can also result in scrotal hair loss; however, partial or complete regrowth of hair usually occurs once the antineoplastic therapy has been discontinued.

Criteria (as defined by a grading system) for evaluating scrotal alopecia has not been established. Researchers have attributed the impetus for pubic hair restoration in women to be prompted by the psychological stress created by the decreased hair (including a sense of inferiority to the same sex) and the perceived adverse effect the hypotrichosis has on their personal (and sexual) life [6-9]. The level of concern by men regarding the development of hair loss from their scrotum and the demand for treatment of scrotal alopecia remains to be determined.

Potential medical therapy for hypotrichosis of the scrotum could incorporate drugs used for androgenic alopecia. These would include either topical minoxidil or oral finasteride [10]. To date, neither of these agents has been evaluated for the treatment of scrotal alopecia.

Hair transplantation has successfully restored pubic hair loss affecting the mons pubis and labia majora in women. The scalp has typically been the donor site—either using strip excision or follicular unit extraction. Micrografts or follicular units have been used for implantation [6-9]. Hence, similar hair restoration techniques could potentially be used for scrotal alopecia in men; alternatives to using the scalp as the donor site for the hair include harvesting the hair from other body sites, such as the beard, trunk, limbs, axilla, and pubic region [11-12].

New therapies for hair loss in men are also being developed. Platelet-rich plasma, as monotherapy or a component of hair transplant restoration, has been utilized to enhance hair growth in the treatment of androgenic alopecia. In addition, other agents and modalities (such as injectable cytokines, low-level laser therapy, and nutraceuticals) that are demonstrating efficacy in the management of male pattern baldness may also be applicable treatments for scrotal alopecia [12-14].

Scrotal Hypertrichosis

Pubic hair grooming is becoming more prevalent not only in women but also in men [15-16]. Removal of pubic hair in women may be partial or total. Total hair removal has been associated with women who are younger in age, have a sexual status of being partnered (as compared to single or married), have received cunnilingus in the previous month, have closely examined their own genitals in the prior four weeks, and who consider themselves to have a more positive sexual function and genital self-image [17].

Pubic hair grooming in men is also common; indeed, similar to women, it is more frequent in younger men. The reasons for grooming include not only preparation for sexual activity (specifically, for performing and receiving oral sex) but also hygiene, comfort, appearance, and curiosity. The hair above the penis is the most commonly groomed by these men; however, groomers also frequently remove hair from their scrotum and penile shaft [16].

**Figure 3:**
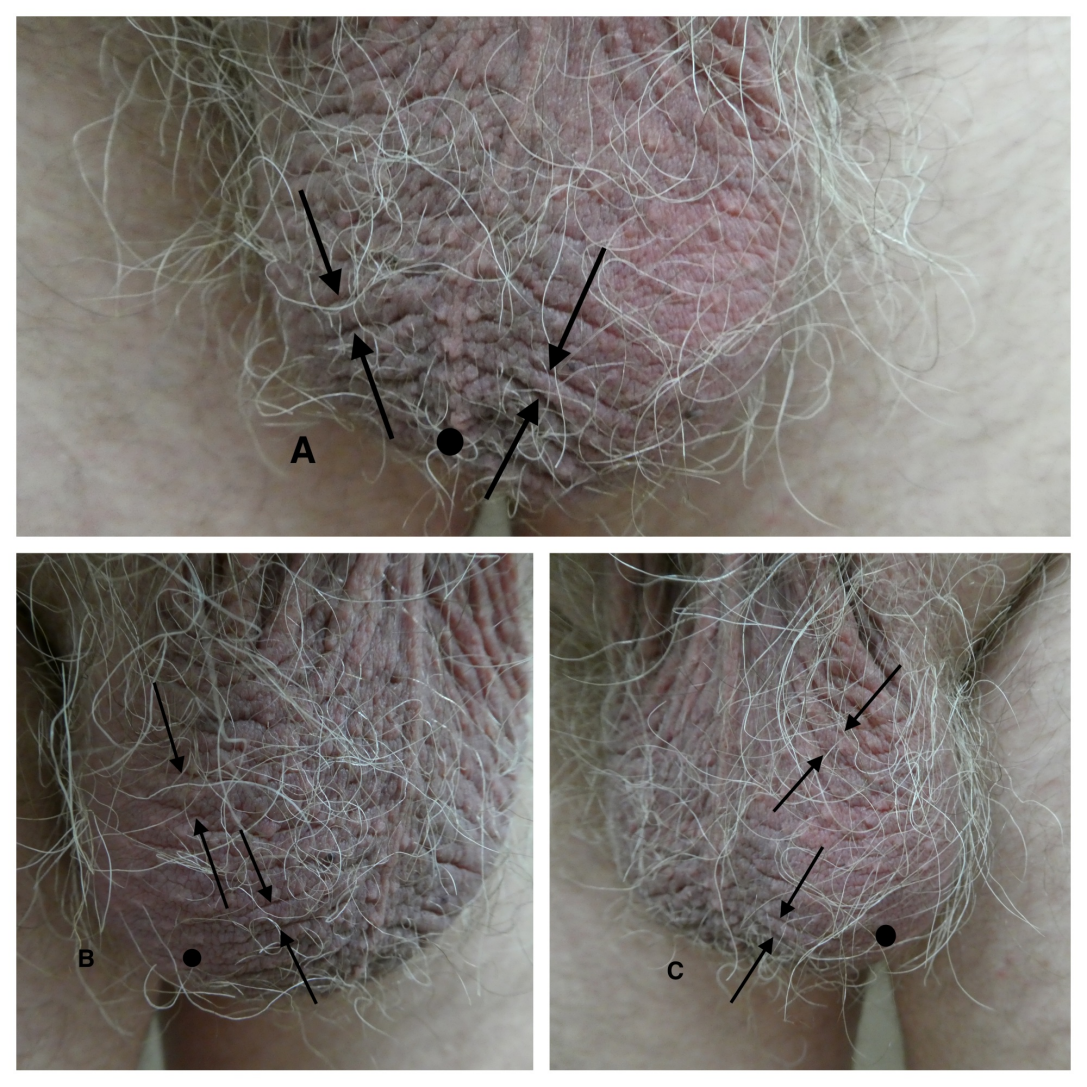
Scrotal hypertrichosis and scrotal wrinkling in a 58-year-old man Frontal (A), right side (B), and left side (C) views of the scrotum (circle) show an increased density of hairs on the scrotum. Scrotal wrinkling (cutis scrotum gyratum) is demonstrated by the prominent folds (between the arrows) and crevices.

Hypertrichosis is characterized by hair growth that is considered to be excessive for the age, race, and race of the affected individual. Although there is a grading scale (the Ferriman-Gallwey classification system) to assess the degree of hirsutism in women, an objective system for evaluating the extent of increased hair growth on the scrotum of men has not been established. Therefore, scrotal hypertrichosis (excessive hair being present on the scrotum) is subjectively determined by the individual’s own perception of normal hair density at that body site (Figure 3).

There are several available methods of hair removal. Shaving, epilation, and depilatories are temporary modalities for removing hair. Shaving (either with a metal blade or an electric razor) is a rapid and simple procedure for the removal of hair in the pubic area of women; however, the skin texture of the scrotum is not readily amenable for this intervention. Similarly, epilation (by plucking or waxing) is also commonly used by women to remove genital hair; yet this method of hair removal is unlikely to be successfully utilized to treat scrotal hypertrichosis [18].

Depilatories—most commonly thioglycolates--are chemicals that can be applied topically to the area of excessive hair growth; within three to 15 minutes, there is the dissolution of the hair shafts. An irritant dermatitis may develop at the location of application; therefore, the depilatory should first be applied to a test site (such as the arm) to ensure that this adverse event does not occur. Albeit uncommon, some individuals may experience an allergic contact dermatitis to the thioglycolate [18].

Permanent hair removal may be achieved with electrolysis, lasers, or intense pulsed light devices. Each of these methods has been successfully used to remove facial hair in women and men; however, multiple treatments are usually necessary [19-20]. Indeed, investigators recently reported successful laser removal of hair growth after six treatments on the scrotal skin of an individual prior to vaginoplasty for male-to-female gender reassignment using a long-pulsed 755-nanometer alexandrite laser at the following parameters: fluences of 18 to 30 Joules per square centimeter, pulse duration of 10 to 20 milliseconds, and spot size of 12 millimeters [21].

Morphology-associated scrotal changes

Scrotal Laxity

Scrotal laxity has also been referred to as a low-hanging scrotum, sagging scrotum, or scrotomegaly (Figure 1). Although laxity of the scrotum may be asymptomatic, it can be a source of embarrassment that prevents the individual from wearing shorts or a bathing suit. However, in some men, the continual rubbing of their scrotum against their thigh causes discomfort. In addition, men with a low-hanging scrotum may experience pain during intercourse or when they exercise and participate in sports.

Scrotal laxity may be related to intrinsic and extrinsic aging. Gravity-dependent stretching of the skin and loss of elasticity are intrinsic factors. Extrinsic factors include previous active involvement in athletics and prior testicular surgery.

Surgical intervention can successfully treat scrotal laxity. The procedure has been described as either a scrotal lift, a scrotal tuck, a scrotoplasty, or a scrotum reduction. The surgery involves removal of excess skin with or without tightening the underlying muscle.

Most of the papers that discuss scrotum reduction surgery describe its use in individuals with either primary (or congenital) or secondary lymphedema of only the scrotum or both the scrotum and penis. However, an internet search of ‘scrotum reduction surgery’ provides several links to websites of physicians who perform this procedure; the sites provide not only additional information about the surgery but also preoperative and postoperative clinical images. One group of physicians briefly discussed the scrotal reduction procedure emphasizing hemostasis (to prevent postoperative hematoma) and preservation of the posterior scrotum (to maintain its superior lymphatic drainage) [4]. Another group of investigators recently shared their surgical protocol (that was used for two adolescent men who had a self-perceived unfavorable appearance of their scrotum) and the favorable results of the researcher’s experience with this surgical intervention [22].

Theoretically, a minimally invasive surgical approach using absorbable suspension sutures might be considered for the treatment of scrotal laxity. Elastic silicone threads have been successfully used to provide functional vaginal rejuvenation [23]. Currently, fully absorbable poly-L-lactic acid (PLLA)/poly-lactide-co-glycolide (PGLA) bidirectional, cone-based, self-anchoring suspension sutures are used for lifting and repositioning facial tissue [24]; modification of not only the suture length but also the cone size, number, and placement might enable this technique to be applicable for providing a scrotal lift.

Scrotal Wrinkling

Scrotal wrinkling can also be referred to as scrotum rugosum or cutis scrotum gyratum (Figures 2, 3). Rugosa (also called tetracorallia) is an order of coral that have wrinkled or rugose walls that line their horn-shaped chamber; the wall of this coral resembles the ridges and furrows observed in men with scrotal wrinkling. Cutis vertices gyrate is a condition characterized by thickening of the scalp with prominent folds and crevices; these skin changes are similar in appearance to those observed in men with wrinkling of their scrotum.

Excessive scrotal wrinkling is a subjective assessment. Skin folds with peaks and valleys typically characterize the normal appearance of the scrotum. Indeed, contraction of the dartos muscle in response to cold temperatures or intercourse may result in the temporary accentuation of scrotal wrinkling.

Botulinum toxin is a neurotoxin; it is produced by the bacterium Clostridium botulinum. Neurotoxin type A preparations are used for therapeutic intervention: abobotulinumtoxinA (aboBoNT/A, ABO, or Dysport), incobotulinumtoxin A (incoBoNT/A, INCO, or Xeomin), onabotulinumtoxinA (onaBoNT/A, ONA, or Botox), and RimabotulinumtoxinB (rimaBoNT/B, RIMA, or Myobloc in the United States or NeuroBoc outside of the United States). Botulinum toxin has been injected into striated muscles of the face to treat facial wrinkling in women and men [25-27].

Botulinum toxin has also been used to treat disorders associated with an aberrant function of smooth muscles [28]. If contraction of the dartos muscle is a contributing component to the etiology of scrotal wrinkling, an intervention directed toward the function of that smooth muscle might result in less wrinkling of the scrotum. Therefore, injection of botulinum toxin into the dartos muscle of the scrotum might result in a smoother scrotal skin surface.

Vascular-associated scrotal changes

Scrotal Angiokeratomas

Angiokeratomas are benign lesions of vascular etiology. They present as purple, red, or black papules; clinically, they can be mistaken for a melanocytic lesion, such as a melanoma. Microscopic examination reveals dilated thin-walled vessels in the upper dermis; hyperkeratosis is typically present in the overlying epidermis.

Angiokeratomas may appear on the skin of the genital region in women (such as the vulva) and men (most commonly on the scrotum, but also the shaft and glans of the penis). When they occur in this area, they are also referred to as angiokeratomas of Fordyce. They may be solitary; however, they usually present as multiple (2 to 5 millimeter) papules (Figure 2) [29-30].

Angiokeratomas of the scrotum are typically asymptomatic. However, bleeding may occur spontaneously or following contact with the lesions following sexual intercourse or excoriation. Indeed, for some men, their scrotal angiokeratomas may cause anxiety, distress, and embarrassment prompting them to seek treatment for the lesions [31].

Destructive modalities are typically used to treat scrotal angiokeratomas. These have traditionally included cryodestruction with liquid nitrogen, curettage, electrocauterization, and surgical excision. More recently, both the 595-nanometer pulsed dye laser and the 1064 nanometer long pulse Nd: YAG laser have been shown to successfully treat these lesions without permanent side effects. Although some men were successfully treated after one session, most individuals required several treatments to achieve complete clearance [32-33]

## Conclusions

Genital rejuvenation includes vaginal rejuvenation in women and scrotal rejuvenation in men. The number of published medical papers on vaginal rejuvenation demonstrates an increased interest in this subject; in contrast, the medical literature does not show a similar awareness of scrotal rejuvenation. Aging and non-age-related changes can occur regarding the hair, the morphology, and the vascularity of the scrotum. Therefore, rejuvenation of the scrotum not only includes conditions for which functional restoration would be appropriate but also issues that may be of significant aesthetic concern to the affected individuals.

Scrotal rejuvenation is characterized by the treatment of hair-associated scrotal changes (alopecia and hypertrichosis), morphology-associated scrotal changes (wrinkling and laxity), and vascular-associated scrotal changes (angiokeratomas). Successful treatments for some of these conditions, such as scrotal hypertrichosis, scrotal laxity, and scrotal angiokeratomas, have already been established. However, some of the therapeutic approaches for alopecia, wrinkling, and sagging of the scrotum are based on modalities that have been used for these conditions when they occur at other body sites, such as the scalp and face.

Medical therapy, such as topical minoxidil and oral finasteride, may be used for scrotal alopecia and conservative modalities, such as depilatories and electrolysis, may be initiated for scrotal hypertrichosis. Scrotal hypertrichosis and scrotal angiokeratomas may be successfully managed with lasers and energy-based devices. The mainstay of therapy for scrotal laxity is surgical intervention; however, it has been hypothesized that an adequate scrotal lift may be provided with the use of absorbable suspension sutures. Postulated treatments for scrotal alopecia and scrotal wrinkling include hair transplantation and the injection of botulinum toxin into the dartos muscle, respectively.

In summary, this paper—to the best of my knowledge—introduces the term ‘scrotal rejuvenation’ into the medical literature. Indeed, scrotal rejuvenation may be able to improve certain cosmetic and functional issues regarding the hair, the morphology and the vascularity of the scrotum. Therefore, as men and their physicians become aware of the conditions of the scrotum that may warrant rejuvenation and the potential treatments of these conditions, the interest in scrotal rejuvenation is likely to increase.
